# Gallein, a Gβγ subunit signalling inhibitor, inhibits metastatic spread of tumour cells expressing OR51E2 and exposed to its odorant ligand

**DOI:** 10.1186/s13104-017-2879-z

**Published:** 2017-10-30

**Authors:** Guenhaël Sanz, Isabelle Leray, Adeline Muscat, Adrien Acquistapace, Tao Cui, Julie Rivière, Silvia Vincent-Naulleau, Valeria Giandomenico, Lluis M. Mir

**Affiliations:** 1 NBO, INRA, Université Paris Saclay, 78350 Jouy-En-Josas, France; 2 Biologie du Développement et Reproduction, INRA, ENVA, Université Paris-Saclay, 78350 Jouy-En-Josas, France; 3 Vectorologie et Thérapeutiques Anti-cancéreuses, UMR8203, CNRS, Univ. Paris-Sud, Université Paris-Saclay, Gustave Roussy, Villejuif, France; 4 Department of Medical Sciences, Endocrine Tumor Biology, Uppsala University, Uppsala, Sweden; 5 GABI, AgroParisTech, INRA, Université Paris-Saclay, 78350 Jouy-En-Josas, France; 6 CEA, DRF, Université Paris-Saclay, 92260 Fontenay-Aux-Roses, France

**Keywords:** Olfactory receptors, β-Ionone, LNCaP cells, Cell invasiveness, Metastasis, Gallein, Gβγ signalling

## Abstract

**Objective:**

We previously reported that the olfactory receptor OR51E2, overexpressed in LNCaP prostate cancer cells, promotes cell invasiveness upon stimulation of its agonist β-ionone, and this phenomenon increases metastatic spread. Furthermore, we showed that the induced cell invasiveness involves a PI3 kinase dependent signalling pathway. We report here the results of a new investigation to address whether gallein, a small inhibitor of G protein βγ subunit interaction with PI3 kinase, can inhibit β-ionone effects both in vitro and in vivo.

**Results:**

We demonstrate that gallein can inhibit the β-ionone-induced cell invasiveness in vitro, as well as the spread of metastases in vivo. LNCaP cell invasiveness, assessed using spheroid cultures in collagen gels in vitro, was increased by β-ionone and the effect was reversed by co-administration of gallein. LNCaP tumour cells, subcutaneously inoculated to immunodeficient mice, generated more metastases in vivo when β-ionone was applied through the skin. Furthermore, the intraperitoneal injection of gallein inhibited this increased metastasis spread. Our results thus support the role of OR51E2 in the β-ionone observed effects, and suggest that gallein could be a potential new agent in personalized medicine of the tumours expressing OR51E2.

**Electronic supplementary material:**

The online version of this article (10.1186/s13104-017-2879-z) contains supplementary material, which is available to authorized users.

## Introduction

Olfactory receptors (ORs) are G protein-coupled receptors (GPCR), first described to sense odorants in the nose [1, 2]. Besides, ORs play additional functions in non-olfactory tissues [3–10]. In particular, they are overexpressed in various tumours, potential tumour markers [11–16] and involved in tumour progression [17–22]. Our previous studies [18, 22] investigated the role of the OR OR51E2, also named PSGR for prostate specific G protein-coupled receptor, which is overexpressed in LNCaP prostate cancer cells. In the presence of β-ionone, an OR51E2 odorant agonist, these cells became more invasive in vitro and generated more metastases in vivo. Since our results were obtained in an androgen-independent context and humans can be exposed to β-ionone through food, beverages and cosmetics, the aggressiveness of prostate tumours expressing OR51E2 could increase due to exposure to β-ionone independently of an androgen receptor stimulation by androgens. Thus, OR51E2 looked to be a potential therapeutic target, and we tried to inhibit it, by using the sole known antagonist, α-ionone. However, we found that α-ionone rather acted as an OR51E2 agonist [22]. We previously demonstrated that a PI3 kinase γ (PI3K γ) dependent signalling pathway is involved in the β-ionone-induced process [18]. Furthermore, gallein, a small molecule that disrupts the interaction of Gβγ subunits with the PI3K γ, was reported to interfere with GPCR signalling in pathologies other than cancer [23–26]. We have now explored whether gallein could also interfere with the OR51E2 receptor signalling involved in cancer progression.

The results reported in this article show that gallein can inhibit the effects of the OR51E2 agonist β-ionone, which promotes cell invasiveness and metastasis dissemination. Thus gallein might be a new anticancer drug to counteract the odorant induced aggressiveness of tumour cells expressing ORs.

## Main text

### Methods

#### Chemicals

The β-ionone, gallein and DMSO were purchased from Sigma-Aldrich and Miglyol 812N from Cremer Oleo Division. Paraffin (CellWax) was from CML. Hemalun, eosin, and saffron were from RAL.

#### Cell culture

LNCaP cells were purchased from ATCC (Clone FGC, No. CRL-1740TM) at passage 20, and grown in RPMI 1640 medium (ATCC) supplemented with 10% fetal bovine serum (ATCC) and 1% penicillin–streptomycin, in a humidified incubator with 5% CO_2_ at 37 °C.

#### In vitro assessment of cell invasiveness

LNCaP cells were grown in 24-well Ultra Low Attachment cell culture plates (Costar) (10000 LNCaP cells per well) to form spheroids. The culture medium was daily renewed. After 4 days, spheroids were transferred into a 28.7% collagen I (Corning) gel according to the protocol described in [27]. Collagen gels were supplemented with β-ionone (1, 10 or 100 µM), gallein 10 µM, or the amount of DMSO (0.1%) used to dilute β-ionone and gallein. 500 µL of the collagen solutions were distributed in wells of a 12-well culture plate and gel solidification was performed 1 h in the incubator. The spheroids were collected and concentrated by sedimentation. The sedimented spheroids were mixed in a proper volume of collagen gel supplemented with β-ionone, gallein or DMSO. 500 µL of the spheroid suspensions were distributed in the collagen-precoated wells. After 1 h in the incubator, 1 mL of culture medium containing β-ionone, gallein or DMSO was added. This medium was renewed daily. Spheroids were followed over time using an Incucyte Zoom instrument (Essen Bioscience) and related software allowed measuring areas of spheroids, released cells or cell clusters. Invasiveness index is the area of migrating cells relative to spheroid area. Three independent wells were used for each condition and five spheroids were followed in each well.

#### Mice

Nod SCID Gamma (NSG) male mice were bred in the animal housing facilities of Gustave Roussy, with free access to food and water. Cages were connected to controlled ventilated racks. The ones with exposure to β-ionone were connected to a distinct ventilation unit. The “Comité d’Ethique en Expérimentation Animale No. 26” approved the study.

#### In vivo assessment of cell invasion and metastases

We used 8–10 castrated male NSG mice (8 week-old) inoculated with LNCaP cells suspended in a mixture of RPMI 1640 and Matrigel (BD Biosciences) (50% each, V/V), for each experimental condition. 10^6^ cells were injected subcutaneously with a 26G needle in each mouse flank. β-ionone (100 µM) was added to the cell suspension for the β-ionone-treated mice only. To this end, β-ionone was first diluted at 100 mM into DMSO, then into the cell-containing mixture. For control groups, the amount of DMSO (0.1%) used to dilute β-ionone was added in the cell suspension. After cell inoculation, a group of mice was treated for 15 days with 1 mM β-ionone in Miglyol applied on skin and a daily intraperitoneal injection of the vehicle used to dilute gallein (1 × PBS, 5% Tween 80, 5% ethanol). Another group was treated for 15 days with 1 mM β-ionone in Miglyol applied on skin and a daily intraperitoneal injection of 5 mg/kg/day of gallein. A first control group received no further treatment. A second control group was treated with Miglyol applied on skin and a daily intraperitoneal injection of the gallein vehicle. A last control group was treated with Miglyol applied on skin and a daily intraperitoneal injection of gallein. Hair was removed on the flanks before cell injection, and every week. Miglyol solution application was performed on the skin above the cell inoculation sites using a sterile cotton swab dipped in the Miglyol solution once per mice. Tumour size was measured with a caliper and tumour volume was calculated as 0.5 × a × b^2^ where a is the longest diameter and b the largest orthogonal diameter. For ethical reasons, mice were sacrificed as soon as the volume of one of the tumours, nodes or metastases reached 1500 mm^3^. Upon autopsy, tumours, lymph nodes, lungs, liver, Tyson glands, stomach, pancreas, spleen and kidneys were sampled. Tissues were fixed for 24 h in 4% formaldehyde, then stored in 70% ethanol at 4 °C. All samples were dehydrated in ethanol and paraffin-embedded. Serial sections of 5 μm thickness were dewaxed in toluene and rehydrated. Some sections were stained with hematoxylin, eosin and saffron (HES staining). Immunohistochemistry was performed on other sections using anti-OR51E2 (1/200, LS-A6332, Cliniscience), anti-PSA (1/100, ab9537, abcam), or rabbit serum as a negative control and antibody fixation was revealed by the Immuno Cruz ABC staining system (Santa Cruz Biotechnology). When at least one metastatic nodule was detected in a tissue section (Additional file 1 for examples), tissue was counted as metastasised. Tissue analyses were performed blindly.

#### Statistical analysis

Data normality was checked using the D’Agostino & Pearson omnibus normality test. To compare metastasis occurence, one way ANOVA was first performed and then all pairs of groups were compared using a two-tailed Student’s test (*p < 0.05). To compare cell invasiveness, a two-tailed Student’s test was performed. To compare Kaplan–Meier survival curves, we used the Mantel-Cox and Gehan-Breslow-Wilcoxon tests.

## Results

### In vitro assessment of cell invasiveness in the presence of β-ionone and gallein

**Fig. 1:**
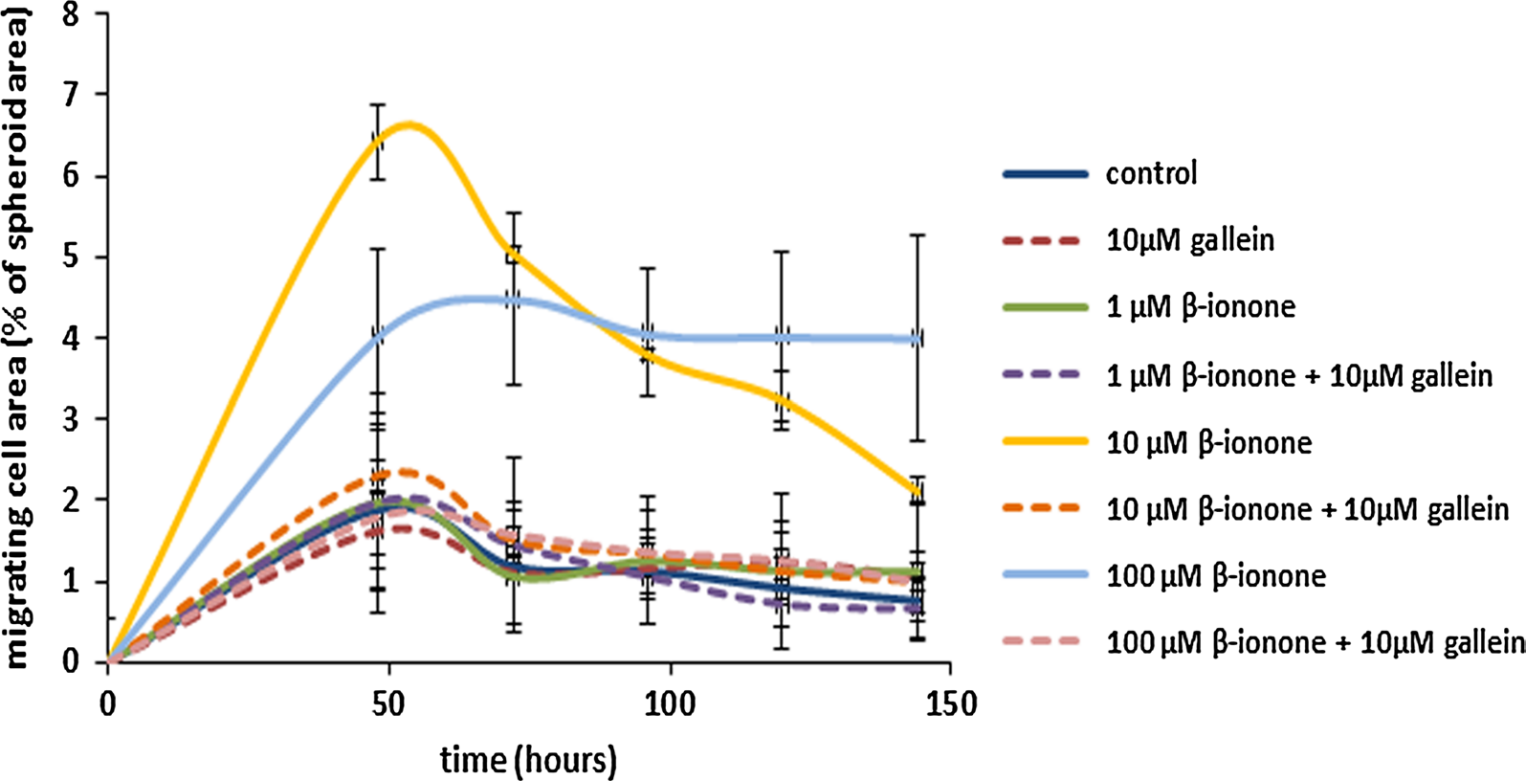
In vitro β-ionone-induced LNCaP cell invasiveness and its inhibition by gallein. Spheroids obtained by using LNCaP cells were grown into a collagen I gel in the presence of various concentrations of β-ionone or of 0.1% DMSO, with (dashed lines) or without gallein. Bars indicate standard deviation between wells corresponding to the same condition (n = 3). Migrating LNCaP cell area relative to spheroid area (%) is shown over time. Compared to the control condition (without β-ionone nor gallein), cell migration from the spheroids was significantly increased (two-tailed Student’s test, p ≤ 0.05) in the presence of 10 or 100 µM β-ionone without gallein, from 48 to 144 h of culture

In the present study, our goal was to establish whether gallein, an inhibitor of the interaction between Gβγ subunits and PI3K, could prevent the β-ionone impact on cell invasiveness. To assess cell invasiveness mimicking a tumor, spheroids made by LNCaP cells were grown in collagen gels and exposed to various β-ionone concentrations, in the presence or absence of 10 µM gallein, previously shown to inhibit the LNCaP cell invasiveness induced by 100 µM β-ionone in another assay [18]. As shown on Fig. 1, β-ionone was able to increase cell migration from spheroids at the concentration of 10 and 100 µM, with a lengthened effect at 100 µM. Gallein was able to significantly counteract this β-ionone-induced LNCaP cell invasiveness.

### In vivo assessment of metastasis occurrence in LNCaP tumours exposed to β-ionone and potential gallein inhibiting effect

We then explored the potential inhibitory effect of gallein on the in vivo β-ionone-induced metastasis spread. Castrated male NSG mice carrying subcutaneous injected LNCaP cells were exposed to cutaneous application of β-ionone twice a day for 15 days, and/or received a daily intraperitoneal injection of gallein at the concentration of 5 mg/kg. Three different controls were included as detailed in Methods: untreated mice and mice exposed to the vehicles used to deliver β-ionone or gallein.

**Fig. 2:**
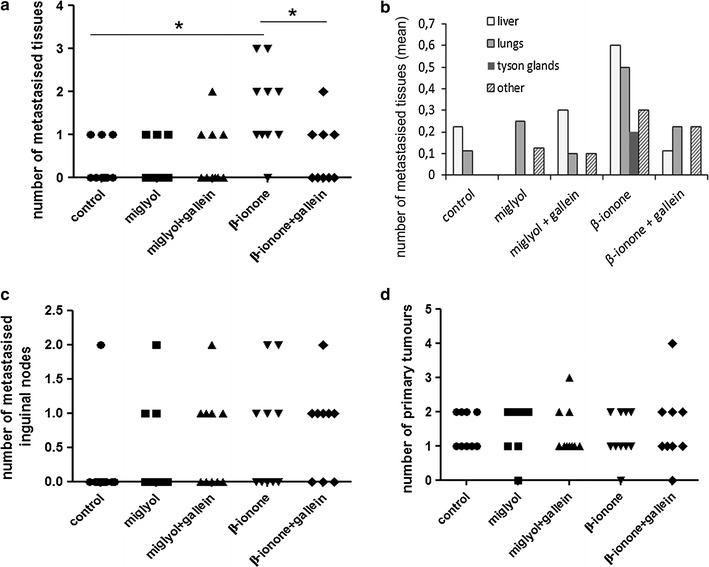
In vivo β-ionone-induced metastasis emergence and its inhibition by gallein. LNCaP cells were subcutaneously implanted in NSG mice. Miglyol or β-ionone diluted in Miglyol was applied on mice skin, while gallein was delivered intraperitoneal. Mice were sacrificed as soon as one of the tumours, nodes or metastases reached 1500 mm^3^. Metastases originating from inoculated LNCaP cells were searched in various tissues by HES staining and immunohistochemistry using anti-PSA and anti-OR51E2 antibodies. **a** Cumulated number of metastasised tissues (that is the number of sampled tissues carrying metastases, not taking into account the inguinal nodes) in each animal of each group. For each mice group, data normality was checked using the D’Agostino & Pearson omnibus normality test. One way ANOVA showed a significant difference between groups and all pairs of groups were compared using a two-tailed Student’s test (*p ≤ 0.05). **b** Mean number of metastasised tissues for each group of mice and for each analyzed tissue. “Other” refers to tissues such as pancreas, spleen, stomach or kidneys. **c** Number of invaded inguinal nodes in each mouse for each group of mice. **d** Numbers of primary tumours

**Fig. 3:**
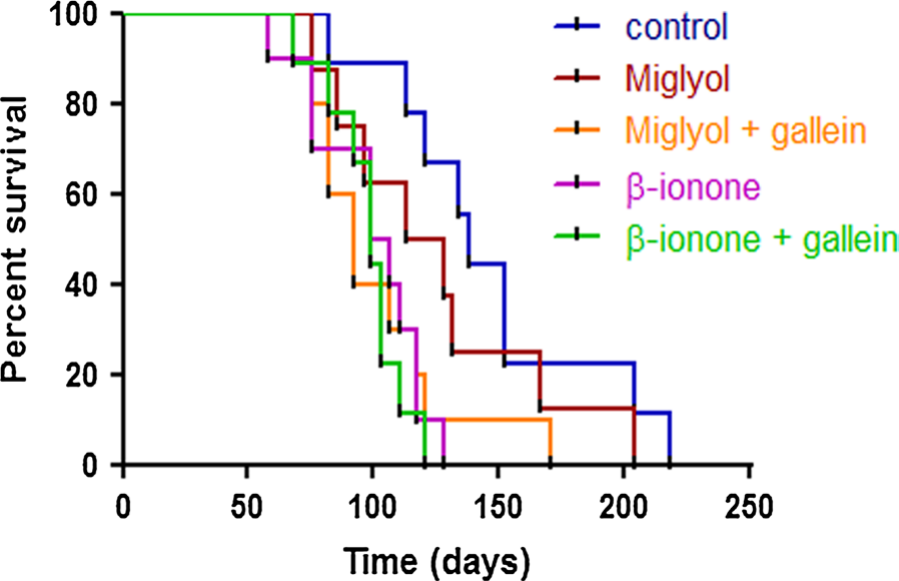
Kaplan–Meier survival curves. Mice were inoculated with LNCaP cells and tumour size was measured as described in the methods section. For ethical reasons, mice were sacrificed as soon as one of the tumours reached 1500 mm^3^. The survival time thus represents the time that the largest tumour took to reach 1500 mm^3^. The Mantel-Cox and Gehan-Breslow-Wilcoxon tests were used to compare survival curves. Only the control and the β-ionone treated groups were significantly different (Mantel-Cox: p = 0.0014; Gehan-Breslow-Wilcoxon: p = 0.0039)

Our results (Fig. 2a, b when the tissues are considered all together) confirm the ones previously obtained showing the β-ionone promoted metastasis emergence [18, 22]. Of major interest is that the gallein’s treatment inhibits the β-ionone effects. We show that the number of metastasised tissues was significantly decreased by the treatment with gallein and the number of metastasised tissues in the group exposed to β-ionone and treated with gallein was similar to the one obtained in the various control groups (Fig. 2a). Nevertheless, the number of β-ionone-induced inguinal metastases was not reduced by gallein (Fig. 2c). Moreover, the “primary” tumours, located at the inoculation sites, did not show any significant difference among the various groups in terms of engraftment (Fig. 2d). Also, confirming previous results [18, 22], the β-ionone-treated group exhibited a slightly faster tumour growth, although the differences in tumour growth rate were not statistically significant (survival cures in Fig. 3—the survival time representing the time that the largest tumour took to reach 1500 mm^3^, at which time mice were euthanised). The treatment with gallein was not able to counteract this slight increase in the “primary” tumour growth induced by β-ionone.

## Discussion

In the present study, we confirm that β-ionone, an OR51E2 agonist, promotes the invasiveness of OR51E2 expressing cells in vitro and their ability to generate metastases in vivo.

Although our in vivo approach, where 1 mM β-ionone was applied on mice skin, cannot measure how much β-ionone reached LNCaP cells, we only presume that it is a small fraction of the applied dose. Since β-ionone is extensively found in food, beverages and cosmetics, humans can be exposed to β-ionone in a bigger amount than our mice, and our results might be relevant for human health.

Furthermore, we show that the β-ionone effects on LNCaP cells are inhibited, both in vitro and in vivo, by gallein which interferes with the signalling pathways induced by GPCRs and involving PI3K. All together, these results support the involvement of a GPCR activated by β-ionone, which is the OR51E2 receptor in our opinion.

Besides, β-ionone appeared to slightly favor LNCaP tumour growth, while gallein was not able to counteract this effect. Thus, the β-ionone promotion of LNCaP tumour growth might involve a different signalling pathway from the one involved in the β-ionone promotion of LNCaP tumour cell invasiveness and propensity to generate metastases. We therefore speculate that β-ionone might induce different signalling pathways when interacting with OR51E2, as the α-ionone does [22].

To conclude, the present results suggest that gallein might be a potential new anticancer drug to counteract the β-ionone-induced metastasis emergence from tumours expressing OR51E2. Yet, gallein does not counteract tumor growth and should be used in combination with other drugs. Moreover, in our study, control mice never exposed to β-ionone and receiving intraperitoneal injections of gallein once a day for 15 days, did not show any adverse effect. In addition, other studies using gallein in mice [23, 24, 28] already showed the absence of adverse effects. Thus, we aim at providing further studies to investigate whether gallein might be used in the treatment of human tumours expressing ORs without any negative side effect.

## Limitations

The main limitation of our work is that we have not fully demonstrated that β-ionone effects are only attributable to OR51E2. A transient or permanent inhibition of OR51E2 in LNCaP cells would be needed. For the moment, we have not found any OR51E2 antagonist, since we showed that the only reported antagonist, α-ionone, is actually an agonist [22]. Regarding OR51E2 knockdown, silencing OR51E2 using siRNAs in pulmonary cells decreased both their proliferation and resistance to apoptosis [29]. Thus, before using an approach to silence OR51E2 which could allow establishing a stable cell line, we first used the validated siRNA to check whether growth and survival of LNCaP cells might be impaired by OR51E2 silencing. Since this was the case, establishing an LNCaP cell line that does not express OR51E2 appeared impossible.

## Additional file

**Additional file 1.** Metastasised tissues showing OR51E2 expression by the metastatic cells.

## Abbreviations

OR: olfactory receptor
GPCR: G protein-coupled receptor
PSGR: prostate specific G protein-coupled receptor
PI3K γ: PI3 kinase γ
NSG: Nod SCID Gamma
