# Supplementary figures and images for: Gallein, a Gβγ subunit signalling inhibitor, inhibits metastatic spread of tumour cells expressing OR51E2 and exposed to its odorant ligand
Source: BMC Res Notes. 2017 Oct 30;10:541. doi: 10.1186/s13104-017-2879-z (PMC5663063; doi:10.1186/s13104-017-2879-z)

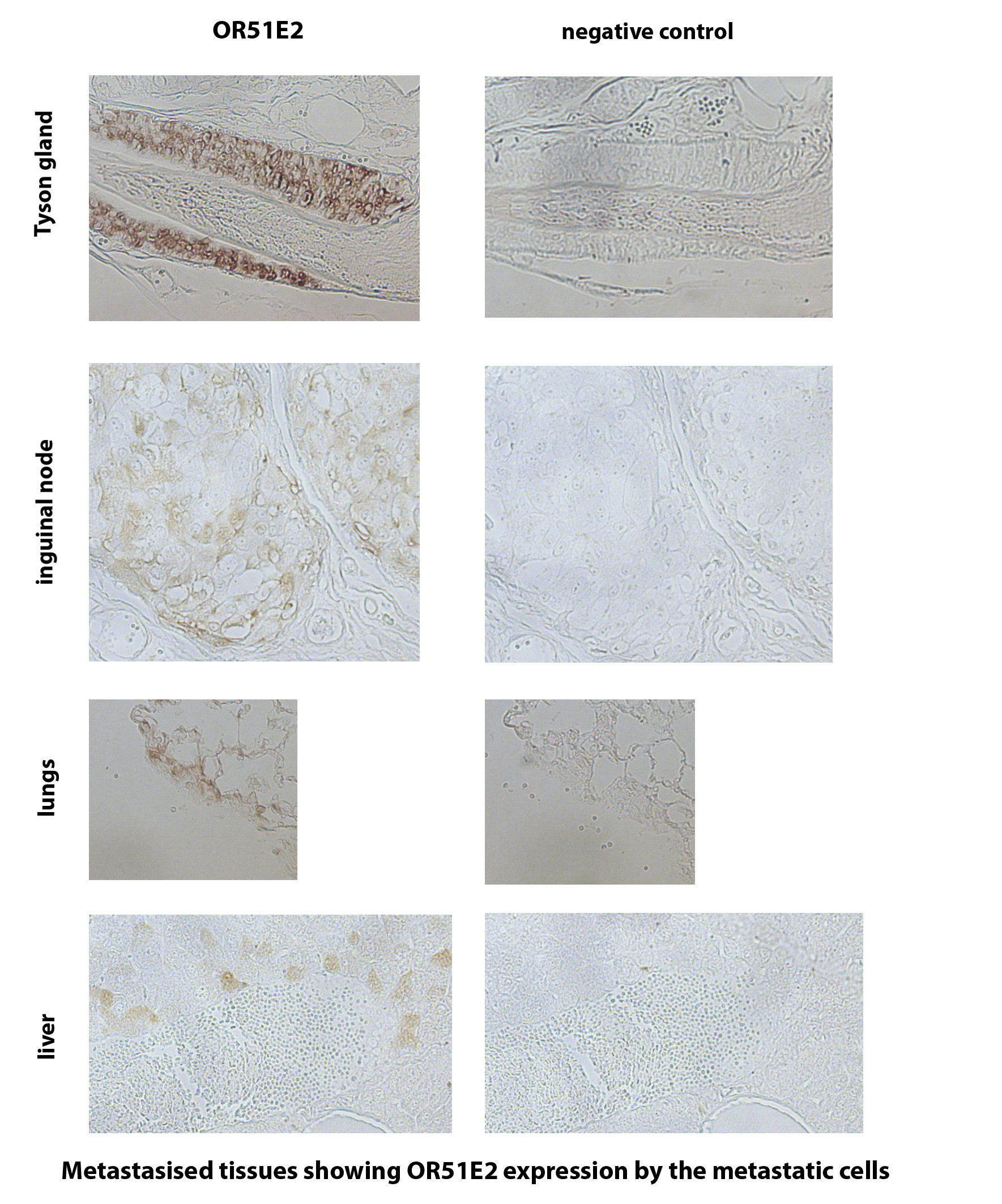

Supplement: Supplementary file 1 — Additional file 1. Metastasised tissues showing OR51E2 expression by the metastatic cells. [file 13104_2017_2879_MOESM1_ESM.tif]
